# Gut Microbiota Profile in Adult Patients with Idiopathic Nephrotic Syndrome

**DOI:** 10.1155/2021/8854969

**Published:** 2021-02-18

**Authors:** Hanchang He, Minwa Lin, Lu You, Tongqing Chen, Zijie Liang, Dao Li, Chao Xie, Guanqing Xiao, Peiyi Ye, Yaozhong Kong, Youlian Zhou

**Affiliations:** ^1^Department of Nephrology, The First People's Hospital of Foshan, Guangdong Province, Foshan 528000, China; ^2^Department of Medicine, Beth Israel Deaconess Medical Center, Harvard Medical School, Harvard University, Boston, Massachusetts 02115, USA; ^3^Department of Gastroenterology, Guangzhou Digestive Disease Center, Guangzhou First People's Hospital, School of Medicine, South China University of Technology, Guangzhou 510180, China

## Abstract

**Background:**

Increasing evidences have reported gut microbiota dysbiosis in many diseases, including chronic kidney disease and pediatric idiopathic nephrotic syndrome (INS). There is lack evidence of intestinal microbiota dysbiosis in adults with INS, however. Here, we to address the association between the gut microbiome and INS.

**Methods:**

Stool samples of 35 adult INS patients and 35 healthy volunteers were collected. Total bacterial DNA was extracted, and the V4 regions of the bacterial 16S ribosomal RNA gene were sequenced. The fecal microbiome was analyzed using bioinformatics. The correlation analysis between altered taxa and clinical parameters was also included.

**Results:**

We found that microbial diversity in the gut was reduced in adult patients with INS. *Acidobacteria*, *Negativicutes*, *Selenomonadales*, *Veillonellaceae*, *Clostridiaceae*, *Dialister*, *Rombousia*, *Ruminiclostridium*, *Lachnospira*, *Alloprevotella*, *Clostridium sensu stricto*, *Megamonas*, and *Phascolarctobacterium* were significantly reduced, while *Pasteurellales*, *Parabacteroides*, *Bilophila*, *Enterococcus*, *Eubacterium ventriosum*, and *Lachnoclostridium* were markedly increased in patients with INS. In addition, *Burkholderiales*, *Alcaligenaceae*, and *Barnesiella* were negatively correlated with serum creatinine. Blood urea nitrogen levels were positively correlated with *Christensenellaceae*, *Bacteroidales_S24.7*, *Ruminococcaceae*, *Ruminococcus*, and *Lachnospiraceae_NK4A136*, but were negatively correlated with *Flavonifractor_plautii* and *Erysipelatoclostridium_ramosum*. *Enterobacteriales*, *Enterobacteriaceae*, *Porphyromonadaceae*, *Escherichia/Shigella*, *Parabacteroides*, and *Escherichia_coli* were positively correlated with albumin. Proteinuria was positively correlated with *Verrucomicrobia*, *Coriobacteriia*, *Thermoleophilia*, *Ignavibacteria*, *Coriobacteriales*, *Nitrosomonadales*, *Coriobacteriaceae*, and *Blautia*, but was negatively correlated with *Betaproteobacteria*, *Burkholderiales*, and *Alcaligenacea*e.

**Conclusion:**

Our findings show compositional alterations of intestinal microbiota in adult patients with INS and correlations between significantly altered taxa and clinical parameters, which points out the direction for the development of new diagnostics and therapeutic approaches targeted intestinal microbiota.

## 1. Introduction

Idiopathic nephrotic syndrome (INS) is a prevalent renal glomerular disorder characterized by edema, heavy proteinuria, and hypoalbuminemia. INS is a substantial cause of pediatric glomerulopathy and is responsible for about 15–30% of adult glomerulopathies [1]. However, the pathogenic drivers of INS have not well defined.

There are various histopathological types of INS, including minimal change nephrosis (MCN), membranous nephropathy (MN), focal segmental glomerulosclerosis (FSGS), mesangial proliferative glomerulonephritis (MsPGN), and membrane proliferative glomerulonephritis (MPGN). Children and adolescents are more likely to have nephropathy with minor lesions, while adults with INS most commonly have membranous nephropathy. In China, MsPGN is the more common pathological type of nephrotic syndrome.

The pathophysiology of INS remains unclear. However, recent evidence supports that gut microbiota contribute to various diseases, including immunological disorders and renal diseases [2–4]. Limited data exists regarding the impact of intestinal microbiota on INS. Kaneko et al. [5] demonstrated that dysfunctional regulatory T cells (Tregs), resulting from dysbiosis of intestinal microbiota, play a crucial role in the deterioration or development of INS in children. Pediatric patients with relapsing INS presented with dysbiosis of intestinal microbiota (characterized by reduced proportions of butyrate-producing bacteria and decreased fecal butyrate levels) that accompanied by decrease in circulatory Tregs, compared to healthy controls [6, 7]. Despite intestinal microbiota, dysbiosis has been reported in children with INS, and research between intestinal flora and adult idiopathic nephrotic syndrome remains limited.

In keeping with this hypothesis, Zhang et al. [8] recently reported intestinal microbiota dysbiosis in adult MN with INS. However, they did not further study associations between gut microbiota and common clinical characteristics. Furthermore, they only enrolled INS patients with pathological diagnosis of membranous nephropathy. This study explores patterns of the intestinal microbiome in adult INS and the association of changes in the intestinal bacterial taxa with common clinical parameters.

## 2. Materials and Methods

### 2.1. Subjects

Subjects consisted of 35 healthy controls and 35 INS in adults. The definition of INS was 24-hour urine protein excretion over 3.5 g along with serum albumin less than 30 g/L based on the Kidney Disease: Improving Global Outcomes (KDIGO) 2012 guidelines [9]. Subjects at age<18 years, with disease caused by secondary factors, treated with corticosteroids or maintenance dialysis, with diabetes or pregnancy, using antibiotics, probiotics, prebiotics, or synbiotics in the previous 4 weeks, or refusing to sign informed consent were excluded. This research received approval of Ethics Committee from the First People's Hospital of Foshan in Foshan, China. All subjects gave a written, signed informed consent.

### 2.2. Collection of Stool Samples and Clinical Parameters

Stool samples were collected by sterile tools and stored at −80°C within 2 hours. Demographic information was collected from medical records. Laboratory data included albumin (ALB), 24 hr proteinuria, serum creatinine (Cr), and blood urea nitrogen (BUN). These data were collected during the first medical care visit. NS patients with pathological diagnoses of membranous nephropathy (MN) or mesangial proliferative glomerulonephritis (MsPGN) were confirmed by the pathologist.

### 2.3. Fecal DNA Extraction, Polymerase Chain Reaction (PCR) Amplification Targeted Bacterial 16S rRNA Genes, Sequencing, and Analysis

Bacterial DNA was isolated from stools using a QIAamp DNA Stool Mini Kit (QIAGEN, Germany) in accordance with the manufacturer's instruction. Concentrations of bacterial DNA were detected with a NanoDrop 2000 BioAnalyzer at 260 nm (Thermo Fisher Scientific, Inc., Massachusetts, United States). DNA specimens were kept under −80°C for further use. PCR and 16S rRNA sequencing were conducted using a MiSeq System (Illumina, Inc.), and the bioinformatic analysis was performed as previously described [10, 11]. Statistical analysis was conducted in Prism GraphPad 6.0 using Student's *t*-test and Wilcox test to compare INS patients vs. control, and MN vs. MsPGN group. Values of *P* less than 0.05 were regarded as statistically significant (^∗^*P* < 0.05, ^∗∗^*P* < 0.01, ^∗∗∗^*P* < 0.005).

## 3. Results

### 3.1. Clinical Characteristics of INS Patients

Clinical data, including sex, age, albumin (ALB), 24 hr proteinuria, blood urea nitrogen (BUN), serum creatinine (Cr), and pathological types, are shown in Table 1. Thirty-five (male : female = 23 : 12) adult patients with INS were enrolled in this study. Average age of onset was 43.40 ± 2.222 years. Mean 24 hr urinary protein levels of 10.91 g, 23.78 g/L ALB, 5.98 mmol/L BUN, and 74.62 *μ*mol/L Cr were also assessed in the INS group. Fifteen patients had membranous nephropathy (MN) while 4 had mesangial proliferative glomerulonephritis (MsPGN).

### 3.2. Diversity of Gut Microbiota in Adult Patients with INS

**Figure 1:**
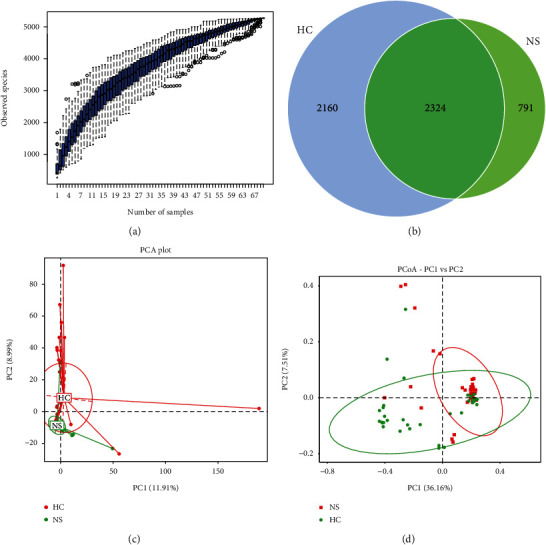
Beta diversity of gut microbiota within healthy controls (HC) and idiopathic nephrotic syndrome (NS, INS) patients: (a) specaccum, (b) Venn diagram, (c) PCA plot, and (d) PCoA plot based on unweighted uniFrac distance.

**Figure 2:**
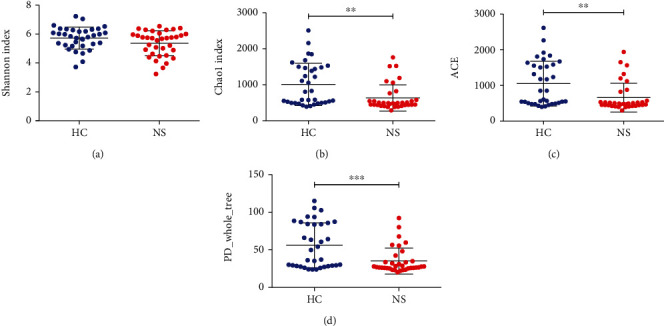
Alpha diversity of gut microbiota within healthy controls (HC) and idiopathic nephrotic syndrome (NS, INS) patients: (a) Shannon index, (b) Chao1 index, (c) ACE, and (d) PD whole tree. Wilcox test, ^∗^*P* < 0.05, ^∗∗^*P* < 0.01, ^∗∗∗^*P* < 0.005.

The species accumulation curve was plotted using specaccum in *R* to show diversity properties of the microbial community (Figure 1(a)). In total, 2,324 OTUs were identified as core microbiota for INS patients and healthy controls (Figure 1(b)). At the genus level, differences in beta diversity were assessed using the unweighted uniFrac distance and visualized with PCoA analysis (Figure 1(d)) and PCA analysis (Figure 1(c)). Alpha diversity showed markedly lower microbial richness (Chao1, ACE, and PD_whole_tree) and Shannon's diversity index for intestinal microbiota in patients with INS compared to healthy controls (Figure 2).

### 3.3. Alterations in the Microbial Structure and Composition in INS

**Figure 3:**
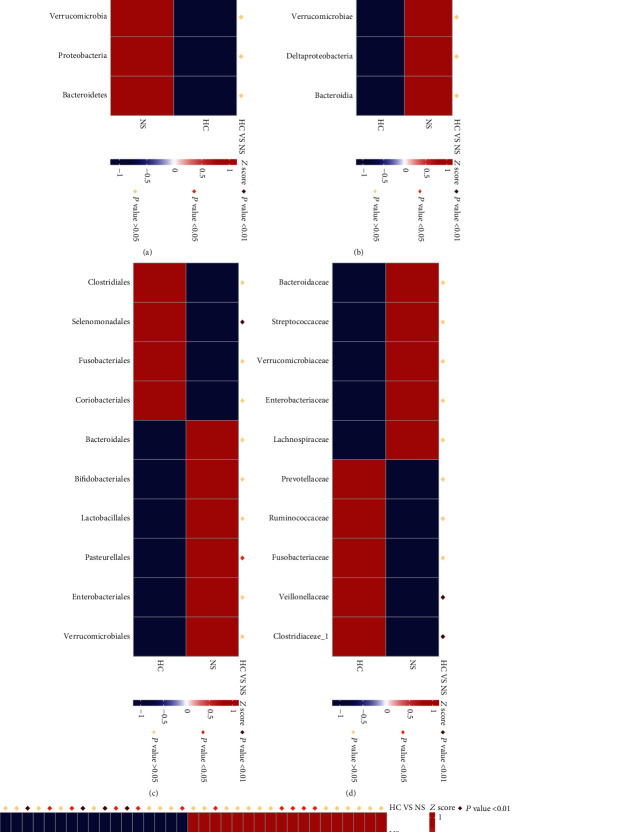
Heatmap of annotation metastat of gut microbiota within healthy controls (HC) and idiopathic nephrotic syndrome (NS, INS) patients: (a) phylum level, (b) class level, (c) order level, (d) family level, and (e) genus level.

**Figure 4:**
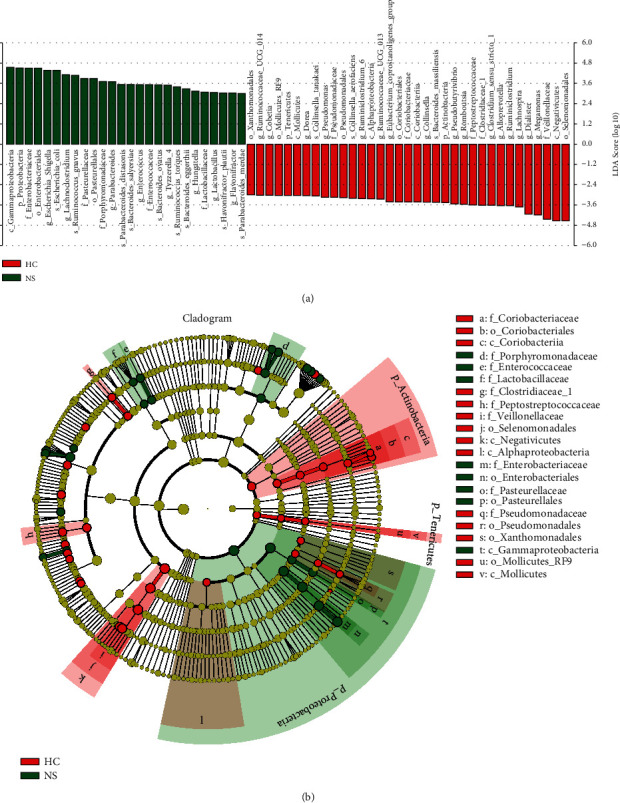
Cladogram generated from linear discriminant analysis (LDA), effect size (LEfSe) (b), and the LDA score (a) showing the most differentially abundant taxa enriched in the microbiota from the healthy controls (HC, red, *N* = 35) and idiopathic nephrotic syndrome (NS, INS, green, *N* = 35) groups. LDA 3.5.

To further explore the fecal microbial difference between INS and healthy controls, we analyzed the intestinal microbiota composition at taxonomic levels. *Acidobacteria* (*P* < 0.01) at the phylum level; *Negativicutes* (*P* < 0.01) at the class level; *Selenomonadales* (*P* < 0.01) at the order level; *Veillonellaceae* (*P* < 0.01) and *Clostridiaceae* (*P* < 0.01) at the family level; and *Dialister* (*P* < 0.01), *Rombousia* (*P* < 0.05), *Ruminiclostridium* (*P* < 0.01), *Lachnospira* (*P* < 0.01), *Alloprevotella* (*P* < 0.05), *Clostridium sensu stricto* (*P* < 0.01), *Megamonas* (*P* < 0.05), and *Phascolarctobacterium* (*P* < 0.05) at the genus level were markedly reduced in patients with INS. *Pasteurellales* (*P* < 0.05) at the order level, *Parabacteroides* (*P* < 0.05), *Bilophila* (*P* < 0.05), Enterococcus (*P* < 0.05), *Eubacterium ventriosum* (*P* < 0.05), and *Lachnoclostridium* (*P* < 0.05) at the genus level were markedly increased in patients with INS (Figure 3). Similar changes in microbial biomarkers were found between patients with INS and healthy controls based on LEfse analysis (Figure 4).

### 3.4. Alterations in Microbial Genera between MN and MsPGN

**Figure 5:**
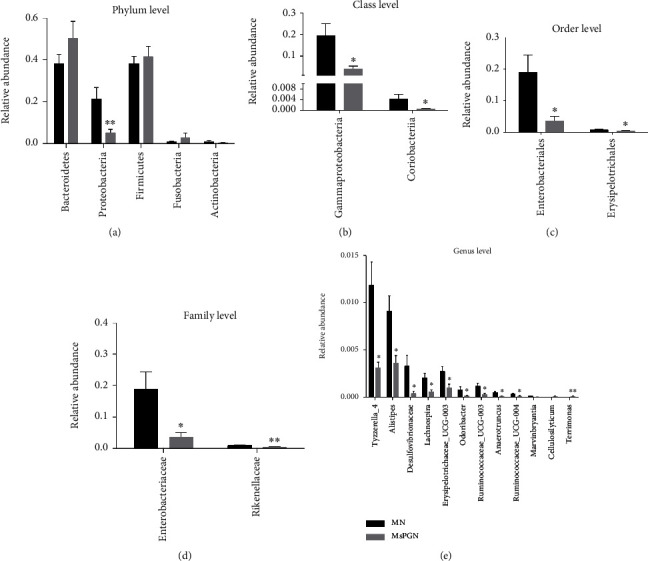
*t*-test bar plot for analysis of bacterial taxa discrepancies between membranous nephropathy (MN) and mesangial proliferative glomerulonephritis (MsPGN) in the phylum level (a), class level (b), order level (c), family level (d), and genus level (e). ^∗^*P* < 0.05, ^∗∗^*P* < 0.01.

Membranous nephropathy (MN) and mesangial proliferative glomerulonephritis (MsPGN) are two of the five histopathological classifications of idiopathic nephrotic syndrome. To distinguish patients with MN from MsPGN and to clarify the role of gut microbiota, we further analyzed the distribution of gut microbiota between these two groups. As shown in Figure 5, many taxa were significantly varied between the two groups. Compared to the MsPGN group, *Proteobacteria* (*P* < 0.01), *Gammaproteobacteria* (*P* < 0.05), *Coriobacteriia* (*P* < 0.05), *Enterobacteriales* (*P* < 0.05), *Erysipelotrichales* (*P* < 0.05), *Enterobacteriaceae* (*P* < 0.05), *Rikenellaceae* (*P* < 0.05), *Chloroplast* (*P* < 0.05), *Tyzzerella* (*P* < 0.05), *Alistipes* (*P* < 0.05), *Lachnospira* (*P* < 0.05), *Odorlibacter* (*P* < 0.05), *Anaerotruncus* (*P* < 0.05), and *Ruminococcaceae_UCG-004* (*P* < 0.05) were increased in MN, while *Rhodobacterales* (*P* < 0.05), *Phyllobacteriaceae* (*P* < 0.05), *Rhodobacteraceae* (*P* < 0.05), *Terrimonas* (*P* < 0.01), and *Mesorhizobium* (*P* < 0.01) (*P* < 0.05) were reduced.

### 3.5. Correlations of Significantly Altered Taxa and Clinical Parameters in INS

To further understand the association between microbial alterations and clinical parameters, Spearman's rank correlation was performed for statistical analysis. As shown in Table 2, Cr had a negative correlation with *Burkholderiales* (*r* = −0.35, *P* = 0.04), *Alcaligenaceae* (*r* = −0.37, *P* = 0.028), and *Barnesiella* (*r* = −0.378, *P* = 0.025). BUN was positively correlated with *Christensenellaceae* (*r* = 0.482, *P* = 0.003), *Bacteroidales_S24.7* (*r* = 0.54, *P* = 0.001), *Ruminococcaceae* (*r* = 0.38, *P* = 0.025), *Ruminococcus* (*r* = 0.34, *P* = 0.045), and L*achnospiraceae_NK4A136* (*r* = 0.38, *P* = 0.023), but was negatively correlated with *Flavonifractor_plautii* (*r* = −0.38, *P* = 0.02) and *Erysipelatoclostridium_ramosum* (*r* = −0.36, *P* = 0.03). ALB was positively correlated with *Enterobacteriales* (*r* = 0.36, *P* = 0.03), *Enterobacteriaceae* (*r* = 0.36, *P* = 0.034), *Porphyromonadaceae* (*r* = 0.36, *P* = 0.036), *Escherichia/Shigella* (*r* = 0.374, *P* = 0.027), *Parabacteroides* (*r* = 0.34, *P* = 0.047), and *Escherichia_coli* (*r* = 0.37, *P* = 0.03). Proteinuria was positively correlated with *Verrucomicrobia* (*r* = 0.36, *P* = 0.03), *Coriobacteriia* (*r* = 0.34, *P* = 0.047), *Thermoleophilia* (*r* = 0.34, *P* = 0.046), *Ignavibacteria* (*r* = 0.37, *P* = 0.03), *Coriobacteriales* (*r* = 034, *P* = 0.047), *Nitrosomonadales* (*r* = 0.38, *P* = 0.03), *Coriobacteriaceae* (*r* = 0.34, *P* = 0.047), and *Blautia* (*r* = 0.39, *P* = 0.02), but was negatively correlated with *Betaproteobacteria* (*r* = −0.36, *P* = 0.03), *Burkholderiales* (*r* = −0.39, *P* = 0.02), and *Alcaligenaceae* (*r* = −0.414, *P* = 0.01).

## 4. Discussion

Several studies have reported the presence of intestinal microbiota dysbiosis in pediatric INS. For example, a reduced abundance of butyrate-producing bacteria such as *Clostridium* clusters IV, XIVa, *Eubacterium* spp., and *Butyrivibrio spp*., and lower concentration of fecal butyrate were confirmed in children [6]. Tsuji et al. [7] further found that pediatric patients in the frequently relapsing INS showed a significant reduction in the proportion of 27 genera (*Propionibacterium*, *Porphyromonas*, *Odoribacter*, *Alistipes*, *Anaerofustis*, *Clostridium*, *Pseudoramibacter*, *Anaerostipes*, *Eubacterium*, *Coprococcus*, *Butyrivibrio*, *Lachnoanaerobaculum*, *Shuttleworthia*, *Roseburia*, *Peptoniphilus*, *Anaerococcus*, *Anaerotruncus*, *Peptoclostridium*, *Faecalibacterium*, *Holdemanella*, *Subdoligranulum*, *Acetonema*, *Acidaminococcus*, *Fusobacterium*, *Megasphaera*, *Brachyspira*, and *Treponema*) to which butyrate-producing bacteria belong. Moreover, 4-week initial therapy, including prednisone and compound of vitamin D3 and calcium, could increase the proportion of short-chain fatty acid- (SCFAs-) producing bacteria including *Romboutsia*, *Stomatobaculum*, and *Cloacibacillus* [12]. Zhang et al. [8] reported the dysbiosis of intestinal microbiome in adult MN.

Our study builds on those finding by demonstrating a decreased alpha diversity (evenness and richness of taxa) in the INS patients compared to healthy controls. A marked bacterial pattern of INS patients was also a reduction of SCFA-producing bacteria, such as reduced abundance of *Romboutsia*, *Clostridium sensu stricto*, *Ruminiclostridium*, and *Lachnospira,* which is consistent with the previous report [8], indicating that a decrease in SCFA-producing bacteria may be the major feature of gut microbiota dysbiosis in both children and adult INS population. In addition to the similar alteration in SCFA-producing bacteria between the children and adult INS population, there are some microbial community changes in the adult INS in the present study. For instance, *Pasteurellales*, *Parabacteroides*, *Bilophil*, Enterococcus, *Eubacterium ventriosum*, and *Lachnoclostridium* were markedly increased in patients with INS. Moreover, we identified changes in the microbial community between subjects with membranous nephropathy and mesangial proliferative glomerulonephritis (MsPGN). The differences in these taxa may serve as biological indicators. We also discovered that certain bacteria were markedly related to common clinical parameters, including albumin, proteinuria, serum creatinine, and blood urea nitrogen levels. Our study first presents an association between altered taxa and common clinical parameters in adults with INS.

Nephrotic syndrome is characterized by heavy proteinuria and hypoproteinemia. Our findings show an association between these clinical parameters and certain bacterial taxa. For example, serum creatinine was negatively correlated with *Burkholderiales*, *Alcaligenaceae*, and *Barnesiella*. Proteinuria was positively correlated with *Verrucomicrobia*, *Coriobacteriia*, *Thermoleophilia*, *Ignavibacteria*, *Coriobacteriales*, *Nitrosomonadales*, *Coriobacteriaceae*, and *Blautia*, but was negatively correlated with *Betaproteobacteria*, *Burkholderiales*, and Alcaligenaceae. These taxa are potential markers for clinical assessment of INS. However, the functions of these bacteria are not yet clear and require further study.

The intestinal microbial community is similarly altered in people with chronic kidney diseases (CKD). *Blautia* was shown to contribute to the difference in gut microbiota of CKD rats compared with sham rats [13]. Among alterations in the gut microbiota and the biochemical parameters of CKD rats, *Blautia* has a positive correlation with the proteinuria level, regardless of systolic blood pressure and creatinine clearance [13], which was consistent with the results of our study.

It remains unclear whether imbalance of intestinal microflora is a consequence or a cause of INS. On one hand, Gut microbiota have been shown to influence INS development, which is alleviated by enhanced Treg activity. Vaziri et al. found that levels of tight junction proteins (such as ZO-1, claudin-1, and occludin) are excessively decreased in the colonic mucosa of animals with chronic kidney disease [14]. A damaged gut barrier enables the transmission and accumulation of uremic toxins, due to a compromised excretory process [15]. On the other hand, factors induced by kidney dysfunction, such as diet restriction, changes in the gastrointestinal biochemical environment, decreased colonic transit and the use of certain drugs such as antibiotics, probiotics, and iron-containing compounds, can also contribute to gut microbiota dysbiosis in patients with CKD [16]. Therefore, the kidney-gut axis might influence the onset, development, and prognosis of INS.

When the diversity, composition, and structure of the intestinal microflora change, microorganisms and antigens activate the host immune system resulting in an inflammatory response [17]. Immune cells are also activated by microbial metabolites and other specific components, thereby exacerbating inflammatory responses and accelerating the progression of kidney disease [18]. For example, Treg cells are induced by SCFAs that are produced by bacteria. Tsuji et al. [6] suggested that gut microbiota dysbiosis involving decreased butyric acid-producing bacteria causes the defects in both induction and differentiation of peripheral inducible Tregs, which then leads to INS relapse.

A systematic review [19] including 23 studies with more than 170,000 patients from 15 provinces/cities in China reported that the top five types of primary glomerulonephritis were immunoglobulin A nephropathy (24.3%), MsPGN (10.5%), MN (12.6%), MCN (9.8%), and FSGS (4.6%). In the present study, some patients did not receive renal biopsy for histopathological classification and only underwent clinical diagnosis. Of the 35 INS patients enrolled in this study, only 15 had MN and 4 had MsPGN. Although the sample size of this subgroup analysis is small, our data shows that some taxa were significantly different between the two subgroups. *Proteobacteria*, *Gammaproteobacteria*, *Coriobacteriia*, *Enterobacteriales*, *Erysipelotrichales*, *Enterobacteriaceae*, *Rikenellaceae*, *Tyzzerella*, *Odorlibacter*, *Anaerotruncus*, *Lachnospira,* and *Ruminococcaceae_UCG-004* were increased in MN, while *Rhodobacterales*, *Phyllobacteriaceae*, *Rhodobacteraceae*, *Terrimonas*, and *Mesorhizobium* were reduced. This variation may indicate new diagnostic biomarkers based on gut microbiota.

Our study also has several limitations. First, the sample size in this study was rather small, especially the subgroups based on the histopathological type. Further, multicenter studies enrolling larger cohort of subjects are needed. Second, due to the lack of follow-up data, we could not evaluate the dynamic shifts in intestinal microflora that may be related to effective therapy. Additionally, we did not explore whether the gut microbial change is a cause or a result of INS. Future research should focus on both mechanistic and translational studies between the gut microbiota and INS, such as microbial metabolites, germ-free mice models, and fecal microbiota transplantation (FMT) from INS patients and healthy donors. Our present study reveals alterations in gut microbiota in adult patients with INS and identifies correlations between significantly altered taxa and clinical parameters in INS. These findings may point out the direction for the development of new diagnostics and therapeutic approaches targeted intestinal microbiota.

## Figures and Tables

**Table 1:** Characteristics of patients with NS and healthy controls.

Characteristic	HC(*N* = 35)	INS(*N* = 35)	*P* value
Age, years, median + SD	38.66 ± 8.15	43.40 ± 13.15	0.074
Gender			0.62
Female	15	12	
Male	20	23	NA
Serum creatinine (Cr, *μ*mol/L)	NA	74.62 ± 27.98	NA
Blood urea nitrogen (BUN, mmol/L)	NA	5.98 ± 3.69	NA
Albumin (ALB, g/L)	NA	23.78 ± 3.9	NA
24 h proteinuria (g)	NA	10.91 ± 4.6	NA
CKD stages			
1	NA	25	NA
2	NA	10	NA
Histopathological types			
MN	NA	15	NA
MsPGN	NA	4	NA
MCN	NA	2	NA
FSGS	NA	1	NA

Note: CKD: chronic kidney diseases; HC: healthy controls; INS: idiopathic nephrotic syndrome; MsPGN: Mesangial proliferative glomerulonephritis; MN: membranous nephropathy; MCN: minimal change nephrosis; FSGS: focal segmental glomerulosclerosis; NA: Not available.

**Table 2:** Correlation analysis of the clinical parameters and taxa relative abundance in the idiopathic nephrotic syndrome group.

Taxa	Serum creatinine (Cr)		Blood urea nitrogen (BUN)		Albumin (ALB)		24 h proteinuria	
	*r*	*P*	*r*	*P*	*r*	*P*	*r*	*P*
Phylum level								
Verrucomicrobia	-0.09	0.60	0.27	0.11	0.28	0.11	0.36	0.03^∗^
Class level								
Coriobacteriia	0.24	0.17	0.02	0.90	-0.06	0.71	0.34	0.047^∗^
Betaproteobacteria	-0.30	0.08	0.08	0.63	0.15	0.38	-0.36	0.03^∗^
Thermoleophilia	0.18	0.30	0.10	0.59	-0.01	0.95	0.34	0.046^∗^
Ignavibacteria	0.09	0.60	0.12	0.48	0.14	0.42	0.37	0.03^∗^
Order level								
Enterobacteriales	0.10	0.59	-0.18	0.30	0.36	0.03^∗^	0.05	0.79
Coriobacteriales	0.24	0.17	0.02	0.90	-0.06	0.71	0.34	0.047^∗^
Burkholderiales	-0.35	0.04^∗^	0.05	0.77	0.15	0.38	-0.39	0.02^∗^
Nitrosomonadales	0.13	0.46	0.25	0.14	0.01	0.95	0.38	0.03^∗^
Family level								
Enterobacteriaceae	0.10	0.59	-0.18	0.30	0.36	0.03^∗^	0.05	0.79
Ruminococcaceae	-0.12	0.49	0.38	0.03^∗^	-0.25	0.14	0.26	0.14
Porphyromonadaceae	-0.13	0.47	0.01	0.96	0.36	0.04^∗^	0.07	0.69
Bacteroidales_S24.7	0.10	0.57	0.54	0.001^∗∗^	0.18	0.29	0.07	0.71
Coriobacteriaceae	0.24	0.17	0.02	0.90	-0.06	0.71	0.34	0.047^∗^
Alcaligenaceae	-0.37	0.03^∗^	0.03	0.86	0.12	0.50	-0.41	0.013^∗^
Christensenellaceae	0.02	0.89	0.48	0.003^∗∗^	-0.01	0.96	0.22	0.20
Genus level								
Escherichia.Shigella	0.07	0.69	-0.29	0.09	0.37	0.03^∗^	0.08	0.65
Ruminococcus	-0.16	0.36	0.34	0.045^∗^	-0.16	0.36	0.25	0.14
Lachnospiraceae_NK4A136	-0.005	0.98	0.38	0.023^∗^	-0.13	0.45	0.33	0.055
Parabacteroides	-0.13	0.46	-0.007	0.97	0.34	0.047^∗^	0.09	0.62
Blautia	-0.006	0.97	-0.05	0.76	-0.18	0.30	0.39	0.02^∗^
Barnesiella	-0.38	0.03^∗^	0.26	0.13	0.13	0.47	-0.04	0.82
Species level								
Escherichia_coli	0.07	0.69	-0.29	0.09	0.37	0.03^∗^	0.08	0.65
Flavonifractor_plautii	-0.02	0.90	-0.38	0.02^∗^	0.31	0.07	0.13	0.45
Erysipelatoclostridium_ramosum	0.10	0.57	-0.36	0.03^∗^	-0.07	0.70	0.10	0.57

## Data Availability

The data used to support the findings of this study are available from the corresponding author upon request.
